# Cross-Modal Data Fusion via Vision-Language Model for Crop Disease Recognition

**DOI:** 10.3390/s25134096

**Published:** 2025-06-30

**Authors:** Wenjie Liu, Guoqing Wu, Han Wang, Fuji Ren

**Affiliations:** 1 School of Transportation and Civil Engineering, Nantong University, Nantong 226019, China; hanwang@ntu.edu.cn; 2 School of Mechanical Engineering, Nantong Institute of Technology, Nantong 226002, China; wgq@ntu.edu.cn; 3 School of Computer Science and Engineering, University of Electronic Science and Technology of China, Chengdu 611731, China; renfuji@uestc.edu.cn

**Keywords:** crop disease recognition, image classification, cross-model data fusion, vision-language model

## Abstract

Crop diseases pose a significant threat to agricultural productivity and global food security. Timely and accurate disease identification is crucial for improving crop yield and quality. While most existing deep learning-based methods focus primarily on image datasets for disease recognition, they often overlook the complementary role of textual features in enhancing visual understanding. To address this problem, we proposed a cross-modal data fusion via a vision-language model for crop disease recognition. Our approach leverages the Zhipu.ai multi-model to generate comprehensive textual descriptions of crop leaf diseases, including global description, local lesion description, and color-texture description. These descriptions are encoded into feature vectors, while an image encoder extracts image features. A cross-attention mechanism then iteratively fuses multimodal features across multiple layers, and a classification prediction module generates classification probabilities. Extensive experiments on the Soybean Disease, AI Challenge 2018, and PlantVillage datasets demonstrate that our method outperforms state-of-the-art image-only approaches with higher accuracy and fewer parameters. Specifically, with only 1.14M model parameters, our model achieves a 98.74%, 87.64% and 99.08% recognition accuracy on the three datasets, respectively. The results highlight the effectiveness of cross-modal learning in leveraging both visual and textual cues for precise and efficient disease recognition, offering a scalable solution for crop disease recognition.

## 1. Introduction

Agricultural crop diseases are a critical threat to global food security, causing annual yield losses of 10–40% and impacting agricultural economies, particularly in resource-limited regions [1,2]. Climate change further intensifies the need for fast and accurate disease detection by increasing the spread of pathogens and making crops more vulnerable [3]. Modern precision agriculture increasingly relies on sensor-based technologies—such as hyperspectral imaging, drones, and IoT-enabled devices—to monitor crop health in real time. These sensor systems generate vast amounts of multimodal data, including high-resolution images, spectral signatures, and environmental parameters, offering unprecedented opportunities for automated disease diagnosis. However, translating sensor-derived data into actionable insights remains challenging due to the complexity of disease patterns and the need for scalable and interpretable solutions.

Traditional disease identification methods, which depend on manual inspection by agricultural experts, are incompatible with the rapid, large-scale data streams generated by modern sensor networks. Human-driven diagnosis is not only labor-intensive but also subjective, error-prone, and impractical for real-time field monitoring. Deep learning models, particularly convolutional neural networks (CNNs), have demonstrated success in processing sensor-captured imagery for disease classification. Compared with traditional machine learning methods, these models excel by learning discriminative patterns directly from raw images, thereby eliminating the need for manual feature engineering and achieving superior accuracy across various disease types. Additionally, CNNs can be implemented on edge devices or drones for real-time disease detection under field conditions, facilitating timely intervention and minimizing crop losses through early diagnosis. Although CNNs have significantly enhanced the efficiency of crop disease detection, several limitations still exist that impede its integration into sensor-driven agricultural systems [4,5,6]. For instance, CNNs need large amounts of labeled data, which can be particularly challenging to acquire for rare diseases or underrepresented crops. Meanwhile, they often face difficulties adapting to various sensor types and changing environmental conditions. Furthermore, existing deep learning frameworks often ignore auxiliary data streams, such as textual symptom descriptions, which could enhance the robustness when fused with visual features [7,8].

**Figure 1:**
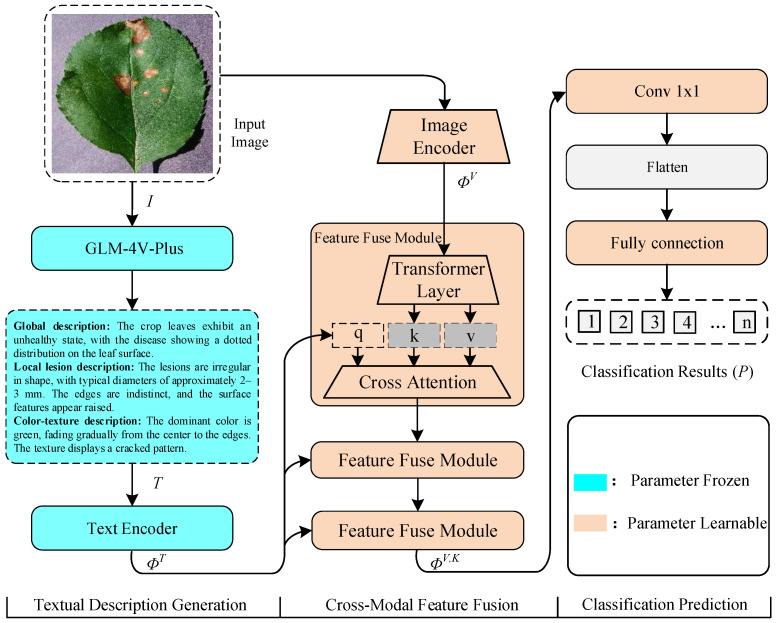
Workflow for our CMDF-VLM, which encompasses three components: text description generation, cross-model feature fusion, and classification prediction.

Therefore, we incorporate the text description features of crop leaf diseases into the model training process, which is expected to effectively guide the model in learning a broader range of crop leaf disease characteristics. Based on this assumption, we propose a multimodal framework specifically optimized for crop disease recognition, called Cross-Modal Data Fusion via Vision-Language Model (CMDF-VLM). As shown in Figure 1, it shows the workflow for CMDF-VLM. The workflow consists of three steps: (1) textual description generation, (2) cross-modal data feature fusion and alignment, and (3) classification prediction. First, the framework generates detailed textual descriptions of the input image using Zhipu.AI’s (Beijing, China) advanced image comprehension model. These descriptions encompass global description, local lesion description, and color-texture description. Second, multiple convolutional layers are utilized to extract and transform visual features from the input image. Meanwhile, the generated text descriptions, encoded into semantic vectors via a text encoder model, are fused with the extracted visual features through multiple consecutive feature fusion modules to realize multimodel data fusion and feature alignment. Third, fused features are used to output classification predictions. By embedding textual information into the model, this approach effectively guides the model to focus on critical areas within crop disease images, thereby improving its recognition performance. Extensive experiments on the Soybean Disease, AI Challenge 2018 and PlantVillage datasets demonstrate the effectiveness of our method. Compared with single-modal CNN, the classification recognition accuracy of the model was significantly improved. In addition, our proposed model framework has fewer model parameters, which is conducive to the accurate identification of crop diseases in resource-limited scenarios.

The main contributions of this paper are summarized as follows:

(1) To solve the multi-modal data fusion of the image and text description features of crop diseases, we propose a cross-modal data fusion framework through a visual-language model. Extensive experiments on multiple crop datasets show that our proposed method can achieve better performance than state-of-the-art methods that rely only on image data to recognize crop diseases.
(2) Compared with other image-only models, our proposed model has smaller parameters and is more suitable for deployment in edge devices to achieve high-precision crop disease recognition.

The remainder of this paper is structured as follows: Section 2 provides a review of related works. Section 3 outlines our proposed methodology. In Section 4, we present and analyze the experimental results obtained from the Soybean Disease, AI Challenge 2018, and PlantVillage datasets. Section 5 evaluates the validity and robustness of our approach. Finally, Section 6 presents our conclusion.

## 2. Related Works

This section reviews the relevant literature on convolutional neural networks for crop disease recognition, multimodal data fusion for crop disease recognition, and vision-language models.

### 2.1. Convolutional Neural Network for Crop Disease Recognition

#### 2.1.1. Traditional CNNs

Deep learning has become a mainstream approach to the identification of crop diseases. Various studies have proposed different models for this task: G. Geetharamani et al. [9] introduced a deep convolutional neural network (CNN) for plant leaf disease detection, while A. Barbedo [10] focused on analyzing individual lesions rather than entire leaves. W. Liu et al. [11] proposed a stochastic channel reuse residual model for crop disease severity detection. J. Chen et al. [12] developed LeafNet, a CNN capable of classifying seven tea leaf diseases, and R. Karthik et al. [13] proposed two deep architectures for tomato leaf infection detection. Y. Zhong et al. [14] designed a DenseNet-121-based model evaluated on an apple leaf dataset with six diseases, and Wagle et al. [15] compared an AlexNet-based network with traditional SVM for disease classification.

#### 2.1.2. Lightweight CNNs

Lightweight models are particularly suitable for deployment in resource-limited environments. General-purpose architectures like ShuffleNet [16], ShuffleNet-v2 [17], MobileNet [18], MobileNet-v2 [19], and GhostNet [20] have shown promise in disease identification but struggle with fine-grained feature extraction. To address this, specialized lightweight models have been developed, such as VGG-ICNN [21] for plant disease recognition and L-CSMS [22] for severity assessment. This paper also aims to propose a lightweight model for plant disease recognition.

### 2.2. Multimodel Data Fusion for Crop Disease Recognition

Recent advancements in multimodal data fusion for crop disease detection have demonstrated significant progress through diverse methodologies. Ametefe et al. [23] propose a synergistic framework integrating deep transfer learning with multimodal techniques to enhance leaf disease detection accuracy through feature extraction and data augmentation. Zhou [24] proposes an “image-text” multimodal and knowledge-assisted model (ITK-Net) for accurate, reliable, and interpretable crop disease identification. Li [25] proposed a multi-source image fusion-based method (AMMFNet) for classifying apple disease and pest areas, achieving higher accuracy and stability by combining RGB and multispectral images with saliency attention and channel attention mechanisms. Lee [26] introduces a deep learning model that uses multimodal data (crop images and environmental variables) for simultaneous crop type prediction, disease detection, and severity assessment, along with a novel multimodal mixup augmentation method, improving disease diagnosis accuracy by 2.58% compared with image-only approaches. Liu [27] introduces the CDDM dataset, a pioneering multimodal resource for crop disease diagnosis, along with a novel finetuning strategy using LoRA to enhance multimodal models, bridging AI advancements and agricultural applications. The above research demonstrates the effectiveness of multimodal data in plant disease recognition and detection. This paper focuses on improving the disease recognition performance of the model by combining disease feature description text generated by a vision-language model with images.

### 2.3. Vision-Language Model

Recently, multi-modal learning that combines visual and language elements has gained significant attention in research [28,29,30]. Notably, vision-language models, such as BLIP [31,32], DALL-E [33,34], GPT4 [35], and Longwriter ZhipuAI [36] have demonstrated impressive performance across various downstream tasks. BLIP excels in bridging pre-trained visual encoders with large frozen language models, offering strong knowledge prompting abilities. The assistance of these large-scale models has shifted focus toward leveraging them to generate detailed natural language descriptions of images. These models supply external common knowledge for image captioning, where key details like dense captions serve as explicit prompts. This allows images to be described comprehensively, capturing essential information effectively. Inspired by this approach, we propose integrating a vision-language model to generate descriptive text for input crop disease images, thereby facilitating the model’s ability to extract features more intuitively and efficiently.

## 3. Methodology

This section details the Cross-Modal Data Fusion via Vision-Language Model (CMDF-VLM), which integrates visual features from crop disease images with semantic textual descriptions to improve classification accuracy. As shown in Figure 1, our method comprises three sequential stages: textual description generation, cross-modal data feature fusion, and classification prediction.

### 3.1. Textual Description Generation

The input disease image *I* is first processed by the GLM-4V-Plus model, which is an advanced image comprehension model developed by Zhipu.AI, to generate hierarchical textual descriptions. The model produces three complementary text components: (1) a global description ($T_{g}$) summarizing the overall distribution of the disease and spatial patterns, (2) a local lesion description ($T_{l}$) detailing fine-grained attributes such as lesion morphology and boundary irregularity, and (3) a color-texture description ($T_{c}$) characterizing chromatic abnormalities and textural properties. The concatenated textual description can be formulated as $T=[T_{g};T_{l};T_{c}]$. Subsequently, *T* is fed into the text encoder of the parameter-frozen BLIP2 [32] model to extract the corresponding text features $\Phi^{T}=\mathrm{Tau}\left(\left[\phi_{g};\phi_{l};\phi_{c}\right]\right)\in R^{d_{t}}$, where $d_{t}$ denotes the dimensionality of the aggregated semantic feature space.

In this stage, the GLM-4V-Plus model and the BLIP2 module do not participate in the model training. Therefore, before training the model, we use the API service of this model from Zhipu.AI to make a call for generating textual descriptions of crop disease images. Then, the locally deployed BLIP2 [32] model is used to convert the description text into feature vectors that can be used for model training.

### 3.2. Cross-Model Feature Fusion

As shown in Figure 1, the step of cross-modal feature fusion contains an image encoder and three successive feature fusion modules. The image encoder, consisting of one 3 × 3 convolution layer, is used to extract and transform the input image features, and it can be represented as follows:

(1) $$\Phi^{V}=F_{conv}\left(I,W_{conv}\right)$$

where $\Phi^{V}\in R^{h\times w\times d_{v}}$, where h×w denotes spatial dimensions, $d_{v}$ the visual feature depth, and $W_{conv}$ refers to the learnable weights of the convolution layers.

Then, three cascaded feature fusion modules align the textual and visual modalities across *K* fusion stages. Each feature fusion module contains a restormer block [37] which is an efficient variant of the Transformer architecture featuring Multi-Directional Transposed Attention (MDTA) and Gated Feed-Forward Networks (GDFN) for hierarchical feature refinement, and a cross attention module. The incorporation of the Restormer block in this study effectively reduces the total number of model parameters while enhancing computational efficiency. In the cross attention module, Key(K) is provided by $\Phi^{T}$, while Value(V) and Qquery(Q) are provided by $\Phi^{V}$. This design utilizes text features as conditions to perform soft filtering on image features, achieving the goal of enabling the crop disease text description feature-guided model to learn. At each stage *k*, the visual features $\Phi^{V,(k-1)}$ are refined using text-guided attention:

(2) $$\Phi^{V,(k)}=\mathrm{Softmax}\left(\frac{Q\left(\Phi^{V,(k-1)}\right)K{\left(\Phi^{T}\right)}^{⊤}}{\sqrt{d}}\right)V\left(\Phi^{T}\right)$$

where *Q*, *K*, and *V* denote learnable linear projections. This process prioritizes disease-related regions described in $\Phi^{T}$, enhancing discriminative feature learning through iterative text-visual interaction.

### 3.3. Classification Prediction

In this step, the probabilities of different classes will be output using the fused features. The fused features $\Phi^{V,(K)}\in R^{H\times W\times D}$ are first processed by a 1 × 1 convolutional layer with *C* output channels to match the number of disease categories, which performs channel-wise dimensionality reduction while preserving spatial information. The convolutional output is then flattened along spatial dimensions and passed through a single fully-connected layer. This design efficiently maps the high-dimensional fused features to class probabilities while maintaining spatial awareness. The final classification probabilities $P\in R^{C}$ are computed as follows:

(3) $$P=\mathrm{Softmax}(W\cdot \mathrm{Flatten}\left({\mathrm{Conv}}_{1\times 1}\left(\Phi^{V,(K)}\right)\right))$$

where *W* represents the learnable weights of the fully connected layer. The model is optimized using standard cross-entropy loss, and the parameters are updated by backpropagation. This streamlined design ensures efficient learning while maintaining discriminative power for disease classification.

### 3.4. Crop Disease Multimodal Dataset Construction

**Figure 2:**
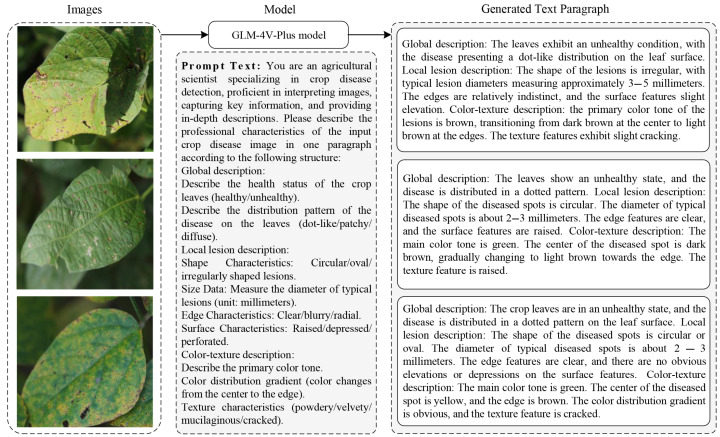
The crop disease multimodal dataset creation process.

**Figure 3:**
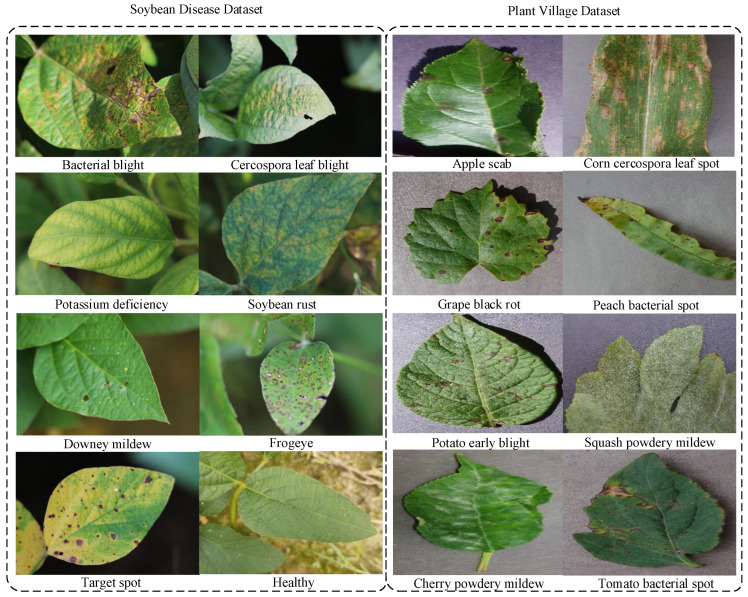
The example images from the Soybean Disease and Plant Village datasets.

We validated our method using three agricultural disease datasets, including the Soybean Disease dataset [38], the AI Challenge 2018 dataset, and the Plant Village dataset [39]. All the images were processed through the GLM-4V-Plus model to generate hierarchical textual descriptions of disease characteristics. Figure 2 illustrates the process of generating textual descriptions for crop disease images using the vision-language model. Crop disease images were fed into the GLM-4V-Plus model, and prompt text was employed to constrain the content generated by the model. Based on the recommendations of agricultural experts, we refined the options in the prompt text to specify the key indicators for crop diseases, thereby enabling the model to produce accurate and usable descriptions of disease features. In the generated text descriptions, we rigorously verified the consistency of the content to ensure that each disease-related image description included three essential components: a global description, a localized lesion description, and a color-texture analysis. For healthy leaves, a standardized description was applied: “Overall observation: healthy leaves; Local lesion description: no visible disease spots; Color and texture analysis: predominantly green”. Thus, we acquired three datasets that include detailed textual descriptions of crop disease characteristics. The Soybean Disease Dataset comprises 10,722 image-text pairs in eight different disease classes, including healthy, bacterial blight, Cercospora leaf blight, downy mildew, frogeye leaf spot, soybean rust, target spot, and potassium deficiency. The dataset is divided into a training set and a testing set in a 7:3 ratio, resulting in 7505 image-text pairs for the training set and 3217 image-text pairs for the testing set. Figure 3 shows the randomly selected images from the Soybean Disease dataset and the Plant Village dataset. The AI Challenge 2018 dataset comprises 35,000 image-text pairs covering 59 categories, including 10 crop types and 27 types of plant diseases. The dataset follows the same 70%–30% split scheme for training and validation. The PlantVillage Dataset comprises 54,305 image-text pairs depicting healthy leaves and disease cases categorized into 36 classes representing 14 crop species. As illustrated in Table 1, the statistical details of the three datasets are provided. We adopted both a standard 7:3 training and testing ratio (38,013 training, 16,292 testing) for comprehensive evaluation. We conducted extensive experiments on these datasets to evaluate our method against state-of-the-art techniques.

## 4. Experiment and Analysis

In this section, we initially introduce the construction methodology and procedural steps of the multimodal crop datasets utilized in our study. Subsequently, extensive ablation studies are conducted across three multimodal crop datasets to evaluate the influence of incorporating text features on model performance. The results are compared with other state-of-the-art methods that rely solely on image-based recognition. Lastly, the Grad-CAM technique is employed for visualization purposes, thereby providing further validation of the proposed method’s efficacy.

### 4.1. Implementation Details

For the Soybean Disease dataset and the AI Challenge 2018 dataset, we adopted standard augmentation techniques during training: (1) random cropping of rectangular regions from square images, with aspect ratios in [3/4, 4/3] and area coverage between [0.08, 1]; (2) resizing the cropped patches to 224 × 224 and (3) applying random horizontal flips and normalization. During evaluation, a 224 × 224 center crop from 256 × 256-resized images was used for classification. For the dataset of the Plant Village, we resized the images to 224 × 224 during the training period only. During the testing period, we employed a configuration similar to that of the Soybean Disease dataset.

In terms of hyperparameter configuration, we employed an initial learning rate of 0.001 with a dynamic decay strategy, reducing it by a factor of 0.5 every 50 training epochs. All the models were optimized using the Adam optimizer for a total of 200 epochs, with a fixed batch size of 64 and a weight decay coefficient of 0.0001. In particular, the entire implementation was developed using the PyTorch 2.1 framework, and all the training procedures were executed on NVIDIA RTX 4090 GPUs (24 GB VRAM), with an AMD 3700 CPU and 64 GB system memory.

### 4.2. Ablation Studies

In order to analyze the impact of crop disease image text descriptions on the model performance, we compared the results obtained after adding different parts of the text descriptions to three crop disease datasets. The textual feature integration framework comprises hierarchical components: global description, local lesion description, and color-texture description. The global description captures overarching semantic context, the local lesion description emphasizes region-specific details, and the color-texture description refines chromatic and structural patterns.

To showcase the effectiveness of these components, we performed a series of ablation studies. The experimental results are reported in Table 2. In the soybean dataset, the baseline model (using images only) achieved an impressive accuracy of 97.99%. By adding global and local texture descriptions step by step, the performance improved further to 98.43% and 98.69%, respectively. When all the textual characteristics were integrated into the complete model, it reached a precision of 98.74%. Similarly, for the AI Challenge 2018 dataset, the baseline accuracy was 85.99%, with incremental improvements to 87.09% (global) and 87.32% (local). The complete model ultimately achieved 87.64%. In addition, for the Plant Village dataset, the baseline accuracy was 97.81%, with incremental improvements to 98.60% (global) and 98.84% (local). The complete model ultimately achieved 99.08%. The experimental results above clearly demonstrate the effectiveness of integrating text features from crop disease images. This approach not only enhances the model’s performance but also highlights the significance of combining all available features to achieve better outcomes.

### 4.3. Image Classification on the Crop Disease Datasets

To demonstrate the effectiveness and adaptiveness of our method, we compared the model performance with other state-of-the-art methods, including ResNet-18 [40], ResNet-34 [40], ResNet-50 [40], MobileNet-v1 [18], MobileNet-v2 [19], GhostNet [20] and ShuffleNet-v2 [17]. Our CMDF-VLM model outperforms other state-of-the-art models in both accuracy and efficiency. On the Soybean Disease dataset, CMDF-VLM achieves a 98.74% accuracy, which is higher than all compared models. At the same time, since the models employed by GLM-4V-Plus and the Text Encoder within this framework are frozen and exclusively used for generating textual descriptions of crop disease characteristics, they do not participate in the model training process. The total number of model parameters corresponds to the learnable parameters involved in the two stages of Cross-Model Feature Fusion and Classification Prediction, as depicted in Figure 1. Consequently, the proposed model comprises only 1.14 million parameters, which makes it significantly more compact compared with other competing models. For example, it has 24.15% fewer parameters than ShuffleNet-v2 but still delivers better accuracy. Similar improvements are seen on the AI Challenge 2018 dataset. Thanks to its lightweight design, CMDF-VLM is ideal for edge devices, where low memory and fast processing are critical. This makes it a practical choice for real-world agricultural applications. Similar to the AI Challenge 2018 and Plant Village datasets, our proposed model also achieves the best accuracy result and fewer model parameters. The consistent performance across datasets confirms CMDF-VLM’s robustness and adaptability to crop disease recognition tasks.

### 4.4. Network Visualization with Grad-CAM

**Figure 4:**
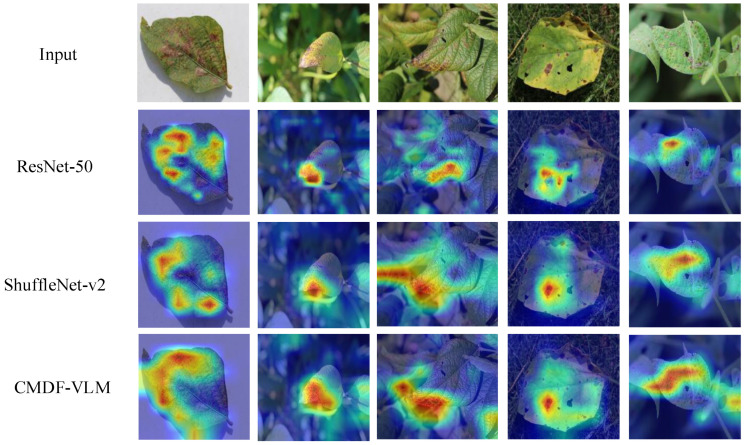
Highlighted the key areas.

To evaluate the effectiveness of our method, we utilized Grad-CAM [41] to emphasize the key areas relevant to our task. Grad-CAM is a visualization technique that employs gradients to determine the significance of spatial locations within convolutional layers. Images were randomly selected from the Soybean disease dataset for this analysis. By highlighting the regions deemed critical by the model for class prediction, we were able to assess the influence of our method on model performance. To examine the impact of our attention module, we compared the visualization outcomes of CMDF-VLM with those of ResNet-50 and ShuffleNet-v2. The results are presented in Figure 4. The heatmap in the figure visually encodes the importance of various regions in the input image for the model’s prediction outcomes using a gradient of colors. Colors closer to red indicate higher levels of importance. It was observed that the highlighted regions produced by CMDF-VLM were more extensive than those from ResNet-50 and ShuffleNet-v2, suggesting that our attention module enables the model to concentrate on a broader range of important areas for the crop disease recognition task. In addition, the heatmap generated by CMDF-VLM covers the location of the crop leaf disease spot well.

## 5. Discussion and Analysis

In this section, we explore the validity of our approach by enhancing feature learning through textual information guidance and improving model parameter efficiency. As shown in Table 2, the accuracy of the model has been effectively improved on the three crop disease datasets. Therefore, it demonstrates the effectiveness of integrating textual guidance into our proposed framework for crop disease recognition.

The proposed framework demonstrates superior performance over other models primarily because it incorporates crop disease description features during training. This integration provides three key advantages: (1) it directs the model’s attention to more disease-relevant features while reducing interference from irrelevant ones; (2) it expands the model’s capacity to learn a wider range of disease-related features and (3) it embeds partial capabilities from advanced image comprehension models, thereby increasing learnable features and boosting overall performance.

Furthermore, the visualization result in Figure 4 shows that textual descriptions guide the model to focus on broader diagnostically relevant regions, enabling it to extract more meaningful features and effectively mimic the decision-making process of agricultural experts. Of course, during the actual deployment process, the image must be passed to advanced image comprehension models to extract disease feature descriptions. These descriptions are then input into the model for disease type prediction. Consequently, the detection process may take slightly longer. However, as shown in Table 3, our proposed framework achieves the best recognition performance with fewer parameters compared with other state-of-the-art models. This makes it highly suitable for scenarios where computing resources are limited yet high recognition accuracy is required. The proposed model can be effectively deployed on edge devices for high-precision detection of crop leaf diseases.

## 6. Conclusions

In this paper, we introduced the Cross-Modal Data Fusion via Vision-Language Model, a framework that improves crop disease classification by combining visual leaf images with AI-generated text descriptions (using GLM-4V-Plus). Our approach leverages hierarchical text descriptions (global, local lesion, and color-texture features) to guide the extraction and fusion of disease-specific visual patterns via cross-modal attention, ensuring better alignment between image regions and diagnostic semantics. Experiments on multiple crop disease datasets demonstrate that CMDF-VLM significantly improves classification accuracy compared with vision-only baselines, particularly in fine-grained disease differentiation. Additionally, our work highlights the potential of vision-language fusion in agricultural AI, where textual descriptions can compensate for visual ambiguities in disease symptoms. Future research will explore real-time deployment and few-shot adaptation for rare diseases. The proposed framework not only advances automated plant disease diagnosis but also provides a reference for multimodal fusion in other precision agriculture applications.

## Figures and Tables

**Table 1:** Statistical summary of Soybean Disease, AI Challenge 2018, and PlantVillage datasets.

Dataset	Number of Images	Number of Categories	Train	Test
Soybean Disease Dataset [38]	10,722	8	7505	3217
AI Challenge 2018 Dataset	35,000	59	24,500	10,500
Plant Village Dataset [39]	54,305	36	38,013	16,292

**Table 2:** Comparison of the image text descriptions impact for model performance.

Dataset	Configurations			Accuracy (%)
	**Global Description**	**Local Lesion Description**	**Color-Texture description**	
Soybean Disease Dataset				97.99
	✓			98.43
	✓	✓		98.69
	✓	✓	✓	98.74
AI Challenge 2018 Dataset				85.99
	✓			87.09
	✓	✓		87.32
	✓	✓	✓	87.64
Plant Village Dataset				97.81
	✓			98.60
	✓	✓		98.84
	✓	✓	✓	99.08

Note: ✓ indicates the module is used in the configuration.

**Table 3:** Comparison of classification accuracy results with other state-of-the-art methods on the Soybean Disease, AI Challenge 2018 and Plant Village datasets.

Description	Soybean Disease Dataset					AI Challenge 2018 Dataset					Plant Village				
	**Params ($\times 10^{6}$)**	**Acc (%)**	**Pre (%)**	**F1 (%)**	**R (%)**	**Params ($\times 10^{6}$)**	**Acc (%)**	**Pre (%)**	**F1 (%)**	**R (%)**	**Params ($\times 10^{6}$)**	**Acc (%)**	**Pre (%)**	**F1 (%)**	**R (%)**
ResNet-18 [40]	11.18	98.05	97.83	97.96	98.11	11.20	86.77	83.54	82.30	82.06	11.19	98.79	98.69	98.70	98.72
ResNet-34 [40]	21.28	97.81	97.60	97.69	97.79	21.31	86.68	83.83	82.58	82.23	21.30	98.65	98.56	98.61	98.66
ResNet-50 [40]	23.52	97.84	97.61	97.72	97.83	23.62	86.92	84.88	83.78	83.82	23.58	98.61	98.55	98.61	98.67
MobileNet-v1 [18]	3.21	97.60	97.41	97.53	97.65	3.26	86.68	85.46	83.97	83.96	3.24	96.32	96.12	96.18	96.25
MobileNet-v2 [19]	2.23	97.71	97.60	97.64	97.69	2.29	86.53	83.95	83.04	83.07	2.27	96.92	96.68	96.70	96.72
GhostNet [20]	3.97	97.25	96.58	96.85	97.16	3.98	86.54	85.91	83.75	83.44	3.98	98.30	98.05	98.07	98.10
ShuffleNet-v2 [17]	4.72	97.95	97.78	97.83	97.89	4.87	85.68	85.02	82.23	81.70	4.81	96.59	96.33	96.42	96.52
CMDF-VLM	1.14	98.74	98.56	98.64	98.72	1.20	87.64	86.18	85.63	85.09	1.17	99.08	98.94	98.98	99.04

## Data Availability

The datasets utilized in this study are openly accessible. The soybean dataset is available at https://doi.org/10.5061/dryad.41ns1rnj3 (accessed on 1 April 2025). The plantvillage dataset is available at https://www.kaggle.com/datasets/abdallahalidev/plantvillage-dataset (accessed on 1 April 2025). The AI Challenge 2018 dataset is available at https://github.com/bochuanwu/Agricultural-Disease-Classification (accessed on 1 April 2025).
